# Academic Performance, Absenteeism, and Drug Use in Emerging Adulthood

**DOI:** 10.7759/cureus.78325

**Published:** 2025-02-01

**Authors:** José Luis Rodríguez-Sáez, Luis Jorge Martín-Antón, Alfonso Salgado-Ruiz, Miguel Ángel Carbonero-Martín

**Affiliations:** 1 Psychology, Universidad de Valladolid, Valladolid, ESP; 2 Psychology, Universidad Pontificia de Salamanca, Salamanca, ESP

**Keywords:** absenteeism, academic performance/grades, drug use, emerging adulthood, emotional distress

## Abstract

This descriptive and cross-sectional study was conducted on a purposive sample of 727 university students, aged 18 to 29 years (M = 21.09, SD = 2.45), grouped based on their psychoactive substance use in the past month. The study aimed to explore drug use and its relationship with academic performance among university students in Castilla y León, Spain. Additionally, it sought to determine whether significant differences in academic performance were associated with potential predictor variables, including absenteeism, substance use, traits of emerging adulthood, and other health-related factors such as depression, anxiety, and stress. The results indicated higher consumption rates of alcohol, tobacco, and cannabis compared to those reported in the Household Survey on Alcohol and Drugs in Spain (EDADES 2022) for individuals aged 15 to 34 in Castilla y León. A relationship between substance use and academic performance was identified, with performance differences observed based on the type of consumer. Although the predictive contribution of the variables included in the regression model was low (10.6%), it remained significant, suggesting the need to refine the model by incorporating additional variables to enhance understanding of the phenomenon. These findings can assist universities in designing preventive interventions.

## Introduction

Drug use poses a serious health problem that increasingly affects young people, particularly when they begin university. The most widely consumed legal substances are alcohol and tobacco, with a concerning rise in binge drinking. The prevalence of binge drinking in the previous 30 days is 15.4%, concentrated among individuals aged 20 to 24 years [1]. Regarding illegal drugs, cannabis is the most commonly used, with first exposure occurring at an average age of 18.3 years. It is also the most widely consumed substance among individuals under 35 years old, with a prevalence of 38.5%.

Among university students, the most commonly used psychoactive substances are alcohol (97.6%), tobacco (72%), and cannabis (55.5%) [2]. The use of these substances is higher among first-year university students than among the general youth population [3]. This consumption, combined with academic stress, negatively impacts mental health [4,5], contributing to issues such as depression, which has a prevalence rate of 55.6% among this group [6].

Several factors may contribute to this worrying trend. First, the concept of emerging adulthood (EA), which spans ages 18 to 29, is characterized by five key features: (1) identity exploration, (2) instability, (3) self-focus, (4) feeling caught between adolescence and adulthood, and (5) experiencing years of exploration [7]. This stage presents challenges such as balancing work and university life, gaining independence, and forming significant new relationships [8]. Second, the university environment imposes multiple responsibilities that increase pressure on students, potentially affecting academic performance [9,10]. Consequences may include failure to complete tasks, absenteeism, and stress [11], which, in some cases, can lead to dropping out of academic activities and turning to substance use as a means of escape [12,13]. However, some authors have found no direct link between academic variables and drug use [14,13].

Absenteeism has been extensively studied, both as a predictor variable for substance use and as a consequence of it [15,16]. It significantly influences substance use among students and is often correlated with increased risk behaviors [15]. Higher levels of substance misuse, particularly involving alcohol and drugs, are associated with increased absenteeism in academic settings [17,2].

This study aimed to expand on previous research by introducing the novel approach of differentiating user groups and incorporating the characteristics of EA. The objective was to develop a more comprehensive understanding of students' behavior during their university years by (1) determining the extent of drug use and its relationship with academic performance among university students and (2) exploring whether significant differences exist in academic performance based on potential predictor variables, including absenteeism, substance use, EA characteristics, and other health-related factors.

## Materials and methods

Study design and procedures

A correlational, cross-sectional study was conducted using a single measure. From April to December 2021, participants were contacted through platforms on the virtual campuses of various universities. Their informed consent was obtained, and they were informed about the study's aim, the voluntary nature of their participation, and the confidentiality of the information being handled. The research ensured data confidentiality following the university's data protection officer guidelines. One of the primary measures adopted was grouping the age variable into ranges rather than using continuous values. This approach reduced the possibility of identifying participants based on their exact age, thereby enhancing anonymity. Furthermore, in compliance with data protection regulations, the records were processed without including personally identifiable information, ensuring that the results could not be linked to specific individuals. The Research Ethics Committee for Medicines - Valladolid Health Area approved the study (approval number: PI 21-2241 NO HCUV).

Sample

A purposive sample of 727 emerging adults (152 males and 575 females), aged between 18 and 29 (M = 21.09, SD = 2.45), was drawn from higher education institutions in Castilla y León, Spain. The sample reflects the actual gender distribution in university degree programs, particularly in fields such as the social sciences, where female enrollment is typically higher. This sampling approach ensures that the study accurately represents the demographic composition of these programs, enhancing its relevance to the target population. While the gender imbalance may limit generalizability to male-dominated fields, it aligns with the study’s focus on understanding behaviors within predominantly female academic environments. The sample’s intentional design thus prioritizes contextual validity over broad representativeness, making the findings more meaningful for the intended academic and demographic context.

To classify the groups, we applied the criterion of inclusion/exclusion based on reported substance consumption over the past 30 days. There were no restrictions on the amounts of substances consumed. The results are shown in Table 1.

**Table 1:** Grouping according to substance use in the last 30 days There were no restrictions on the amounts of substances consumed. l.m: last month (days of consumption in the last month), NCG: non-consumers group, OCG: occasional consumers group, CG: consumers group

Group	N (%)	Nicotine	Alcohol	Cannabis	Other drugs
NCG	121 (16.6)	No	< 2 days/l.m.	No	No
OCG	375 (51.6)	< 9 days/l.m.	> 3 days but < 9 days/l.m.	< 2 days/l.m.	No
CG	231 (31.8)	> 10 days/ l.m.	< 10 days/l.m.	> 2 days/l.m.	Sometimes

Evaluation tools

Academic performance was evaluated using four items. Participants were asked about their average grade/mark, the grades/marks obtained at the end of the academic year, the number of hours they studied per week, and the number of days they missed lessons.

Sociodemographic variables and certain aspects of substance use were assessed using an ad hoc questionnaire already applied in the pilot study conducted by Rodríguez-Sáez et al. [2]. In addition, two specific alcohol and cannabis scales were used: the Alcohol Use Disorders Identification Test (AUDIT-C; [18]), validated by García Carretero et al. [19], and the Cannabis Abuse Screening Test (CAST) [20]. Both instruments are valid and reliable tools for evaluating alcohol and cannabis consumption in young people. They are also easy to administer, facilitating consistent data collection in studies with large samples.

The AUDIT-C was used to identify disorders triggered by alcohol use among the university population. This instrument demonstrates good internal consistency (α = 0.83) and high sensitivity and specificity in detecting risk consumption. In this study, it achieved an α = 0.75. The test consists of three questions related to alcohol consumption problems and is easy to administer. The AUDIT-C cut-off point for identifying risk consumption was five points for males and four for females.

The CAST evaluates cannabis severity and dependence, considering various validity components among adolescents and young adults [21,22]. It demonstrates good internal consistency (α = 0.84). In this study, it achieved an α = 0.89. Individuals who score four or more (as indicated in the Household Survey on Alcohol and Drugs in Spain (EDADES 2022) survey) are considered at high risk of developing problematic cannabis consumption.

The Spanish version by Bados et al. [23] of the short version of the Depression, Anxiety, and Stress Scales (DASS-21) [24] was used. This scale comprises 21 items divided into three subscales of seven items each, assessing depression, anxiety, and stress. Each item describes a negative emotional state experienced over the past week, and participants rate their responses on a four-point Likert-type scale. To categorize symptom severity (i.e., no symptoms, mild, moderate, severe, or extremely severe), we used the cut-off points analyzed by Antony et al. [25]. The test demonstrates good internal consistency for its subscales, ranging between 0.70 and 0.82. In this study, the values ranged between 0.83 and 0.90.

The Spanish version of the Inventory of the Dimensions of Emerging Adulthood (IDEA-S) [26], consisting of 31 items across six subscales, was used. Responses are provided on a Likert-type scale ranging from 1 to 4, with no reversed items. The internal consistency indices obtained for the subscales ranged between α = 0.53 and 0.86. In this study, they ranged between 0.54 and 0.81.

Statistical analysis

As the assumptions of normality required for parametric tests were not met, since the Kolmogorov-Smirnov test for one sample indicated that all variables analyzed in the study had a significantly non-normal distribution (p < 0.001), non-parametric methods were used to ensure adequate statistical analysis. A descriptive analysis of the data was conducted. The Kruskal-Wallis test was applied to assess differences among various consumer groups, academic performance levels, and absenteeism, including the η2H statistic as a measure of effect size: 0.01-<0.06 (small effect), 0.06-<0.14 (moderate effect), and ≥0.14 (large effect). The Mann-Whitney U test was used as a post-hoc analysis with Bonferroni adjustment.

Spearman’s rho (rs) bivariate correlation test was conducted to analyze the relationship between substance consumption frequency and academic performance. Students were categorized based on performance to determine whether significant differences in academic performance existed concerning drug use and other related variables, with a threshold of eight out of ten, given that the participants' academic performance was medium to high (M = 7.2, SD = 0.98). Significant differences were found between bachelor's degree students (Mdn = 7) and master's degree students (Mdn = 8) (z = -3.79, p < 0.001). Finally, binary logistic regression was applied, dichotomizing academic performance to assess whether absenteeism, substance use, and other related variables were significant predictors of academic performance. The statistical analyses were performed using SPSS Statistics version 29 (IBM Corp. Released 2019. IBM SPSS Statistics for Windows, Version 26.0. Armonk, NY: IBM Corp.).

## Results

The most widely used psychoactive substances were alcohol, tobacco, and cannabis. A total of 95.6% (N = 695) of students had consumed alcohol at some point, while 53% (N = 385) had consumed cannabis. Regarding tobacco, 65.9% (N = 479) had tried it, and 18.3% (N = 133) smoked daily. Among smokers, the mean number of cigarettes consumed per day was 7.71 (SD = 4.66). A total of 9.2% (N = 67) had tried tobacco for the first time between the ages of 12 and 13, while 22.7% (N = 165) had done so between the ages of 14 and 15. It was found that 68.4% (N = 497) of the total sample had been drunk at least once over the previous 12 months. Furthermore, 9.6% (N = 70) had been drunk on more than 24 occasions over the past year, while 38.4% (N = 279) reported binge drinking within the preceding two weeks. A total of 17.2% (N = 125) reported having tried alcohol for the first time between the ages of 12 and 13, while 41.3% (N = 300) had done so between the ages of 14 and 15. It was also found that 37% (N = 269) exhibited a risk score on the AUDIT-C. Additionally, 27.9% (N = 203) had consumed cannabis over the past year, while 12.8% (N = 93) had done so within the preceding month. A total of 6.3% (N = 46) showed a risk score on the CAST scale (< 4). Regarding the use of other drugs, consumption was less prevalent.

A correlation analysis was performed to explore the relationships between academic variables and substance use. Table 2 shows that high academic performance was associated with lower tobacco and alcohol consumption over the past year. In terms of absenteeism, higher absenteeism was linked to greater consumption of all substances over the last year except for opiates.

**Table 2:** Correlations between academic variables and consumption in EA Spearman's Rho correlation was calculated considering *p < 0.05, **p < 0.01. EA: emerging adulthood, LSD: lysergic acid diethylamide

	Academic performance	Absenteeism
Tobacco	-0.121^**^	0.258^**^
Alcohol	-0.104^**^	0.287^**^
Cannabis	-0.060	0.282^**^
Cocaine	0.062	0.123^**^
Amphetamines	-0.062	0.094^*^
Sedatives/tranquilizers	-0.007	0.088^*^
Hallucinogens (LSD)	-0.040	0.080^*^
Opiates	-0.012	-0.042
Inhalants	-0.040	0.150^**^
Designer drugs	-0.061	0.115^**^
Other drugs	-0.021	0.114^**^

A link was also found between academic performance and one of the dimensions of EA (instability/negativity): ρ = -0.082, p < 0.05, and between academic performance and depressive symptomatology (ρ = -0.150, p < 0.01). Absenteeism was also found to be related to another dimension of EA (feeling in-between): ρ = 0.073, p < 0.05, as well as between absenteeism and two additional dimensions of emotional malaise: depression (ρ = 0.120, p < 0.01) and anxiety (ρ = 0.099, p < 0.01).

When applying the Kruskal-Wallis test (Table 3), differences were found in academic performance based on whether the student was a consumer (CG), a sporadic consumer (OCG), or a non-consumer (NCG), with an effect size between medium and large. Differences were observed in academic performance between groups (H2(2) = 12.979, p = 0.002, h² = 0.07). Differences also emerged in absenteeism (H2(2) = 50.883, p < 0.001, h² = 0.12). Applying the Mann-Whitney U test for post-hoc analysis using the Bonferroni correction, differences were found between the consumers and the other two groups. CG was found to have the lowest academic performance (M = 7.03), while NCG had the highest (M = 7.36). Differences in absenteeism were also observed between the three groups. CG showed the highest rate of absenteeism (M = 10.25), followed by OCG (M = 4.58), and finally NCG (M = 2.65).

**Table 3:** Kruskal-Wallis test across consumption groups The Kruskal-Wallis statistic and the effect size (eta squared) were estimated. The Mann-Whitney U-test was applied for the post hoc analysis, and the Bonferroni correction was used. ^1,2,3^ Significant difference between groups (in which 1 > 2 > 3), α < 0.017 NCG: non-consumers group, OCG: occasional consumers group, CG: consumers group

	Average range					
	NCG (n = 121)	OCG (n = 375)	CG (n = 231)	X^2^ (2)	p	h^2^_H_
Academic performance	389.30^1^	379.69^1^	325.28^2^	12.979	0.002	0.07
Absenteeism	269.45^3^	353.80^2^	430.08^1^	50.883	< 0.001	0.12

Finally, considering the correlations indicated in Table 2, we calculated the binary logistic regression model that defines the academic performance of young university students (Table 4). This model yielded a Chi-square significance in the omnibus test of p < 0.001, indicating that it appears adequate. However, only 10.6% of the dependent variable could be explained. The Hosmer-Lemeshow test for model goodness of fit resulted in p = 0.495 (p > 0.05), suggesting that the model fits the reality well. The linkage between academic performance as the dependent variable and the rest of the covariates yielded statistically significant differences for absenteeism, instability/negativity, alcohol consumption evaluated using the AUDIT-C, depression, and stress (p = 0.005; p = 0.028; p = 0.002; p < 0.001; p < 0.001), with negative relationships in the first four cases (B = −0.033; B = −0.319; B = −0.122; B = −0.086).

**Table 4:** Binary logistic regression model of academic performance ^a ^Variables specified in step 5 AUDIT-C: Alcohol Use Disorders Identification Test

Academic performance								
Source^a^							95% C.I. for Exp (B)	
	B	S.E.B.	Wald	df	p	Exp (B)	Lower	Upper
Absenteeism	-0.033	0.012	7.994	1	0.005	0.968	0.946	0.990
Instability/negativity	-0.319	0.145	4.819	1	0.028	0.727	0.547	0.966
AUDIT-C	-0.122	0.039	9.985	1	0.002	0.885	0.821	0.955
Depression	-0.086	0.022	14.927	1	0.000	0.917	0.878	0.958
Stress	0.102	0.024	17.718	1	0.000	1.107	1.056	1.161

In absenteeism, the odds ratio (Exp(B) = 0.968, 95% CI (0.946, 0.990), p = 0.005) suggests that for each additional unit of absenteeism, the odds of better academic performance decrease by approximately 3.2% (1 - 0.968). For instability/negativity, with an odds ratio of 0.727 (95% CI (0.547, 0.966), p = 0.028), a higher level of instability/negativity is associated with a 27.3% decrease in the odds of better academic performance. For AUDIT-C, an odds ratio of 0.885 (95% CI (0.821, 0.955), p = 0.002) indicates that higher AUDIT-C scores (alcohol use) reduce the odds of better academic performance by 11.5%. For depression, the odds ratio (Exp(B) = 0.917, 95% CI (0.878, 0.958), p < 0.001) shows that increased depression scores lower the odds of better academic performance by 8.3%. For stress, in contrast to the negative predictors, stress has an odds ratio of 1.107 (95% CI (1.056, 1.161), p < 0.001), meaning that higher stress levels are associated with a 10.7% increase in the odds of better academic performance.

The remaining variables of sex, tobacco smoking, cannabis consumption (CAST), anxiety, and the remaining dimensions of AE were not linked to academic performance.

## Discussion

Although the results from this study are not generalizable to all university students in Castilla y León, they provide an overview of psychoactive substance use in the region, as well as some characteristics of academic performance and their interrelationships. We found higher prevalence rates for alcohol, tobacco, and cannabis among university students compared to the levels reported in the EDADES 2022 survey for the 15- to 34-year-old age range in Castilla y León over the last 12 months [27]: alcohol consumption is 5.1 percentage points higher, tobacco consumption is 5.2 percentage points higher, and cannabis consumption is 13.9 percentage points higher. Furthermore, the frequency of alcohol intoxication among university students over the last 12 months (70.4% for males; 67.8% for females) is much higher than the figures reported by the EDADES 2022 survey (37.6% and 27.2%, respectively). While binge drinking over the last month (29.3% for males and 17.5% for females among university students) is similar to the 31.5% and 18.8% reported in the EDADES 2022 survey, the consumption rate of cannabis over the last 30 days is 7.6 percentage points higher among university students compared to the 15- to 64-year-old sample in Castilla y León. However, it is lower than rates found in other studies involving university student samples [3].

It is important to highlight that those who reported not having consumed any substances had above-average grades. Consequently, students in the GCE group had lower grades, while those in the GC group reported even lower grades. This leads to the conclusion that drug use does indeed impact academic performance, which is consistent with the findings reported by Arellanez-Hernández et al. [28].

Regarding the second research objective, the results align with other studies: absenteeism is a risk factor for poor academic performance. It can be argued that students with better academic performance are more motivated to attend classes and feel more comfortable in an academic environment, which also helps them develop prosocial attitudes that counter drug use [29]. Some studies suggest that substance use can harm young people’s academic performance [9,10,30,31]. Although a correlation was found between tobacco smoking over the previous 12 months and academic performance, this variable did not predict academic performance, as found in other studies [31].

García-Mendoza et al. [32] also tested the link between EA and commitment to studies, finding that most emerging adults showed high commitment to their higher education careers, with a mean score of 8.24 (SD = 1.49). In the present study, the only trait of EA that correlated with academic performance was instability/negativity (ρ = -0.082, p < 0.05). One possible explanation is that at this stage of their lives, students see pursuing a university degree as a means to achieve financial independence and access personal and cultural development. However, it is also a time when they may experience negativity due to dissatisfaction with their degree choice or fears about their ability to graduate.

Several studies have explored the link between pressure from the higher education system and students’ health, demonstrating that stress negatively impacts overall health [33,34], affecting academic performance and quality of life and influencing behaviors such as substance use and academic cheating [35]. A recent study indicates that higher levels of stress lead to lower academic performance (r = -0.60, p < 0.001) [36]. However, the present study yields contradictory results, finding that stress does not correlate with academic performance and that higher stress levels are associated with a 10.7% increase in the odds of better academic performance. Despite the belief that stress negatively impacts academic performance, some studies suggest that effective stress management can lead to better outcomes [37]. This highlights the complexity of the relationship between stress and academic success, indicating that context and coping mechanisms play a crucial role. Additionally, this study found a link between academic stress and depression (ρ = 0.688, p < 0.001). Restrepo et al. [38] support the idea of a shared origin, suggesting that academic stress could be a variable explaining the correlation between substance use and depression.

Limitations

Regarding the limitations of this study, it is important to acknowledge that a non-probability sampling method was used, which limits the generalizability of the findings. As a result, the conclusions are only representative of the specific study sample. For future research, it is suggested that studies utilize a probability sample that includes both public and private universities. This approach would enhance the generalizability of the results and allow for more comprehensive and nuanced analyses by facilitating comparisons across different university types. On the other hand, it is important to consider the high percentage of women in the sample. However, the female population in the universities of Castilla y León is larger than the male population. This could explain why some of the expected results were not obtained since, in general, women have a consumption pattern that is less harmful to health: they consume less frequently and in smaller quantities than men. Additionally, it would be valuable to examine the presence of EA among young people who are not at university and those who are employed or are neither studying nor working. Such research would contribute to a deeper understanding of the variables affecting this relatively understudied group.

Furthermore, our study lacked a comprehensive exploration of the substance use pattern, including critical factors such as age of onset, amounts consumed, duration, reasons for initiation, etc. Incorporating a more detailed assessment could provide deeper insight into the phenomenon of substance use among university students.

Another limitation of the study is the lack of control over factors such as prior mental health status and access to psychological resources. Participants with a history of emotional distress may have reported higher levels of symptomatology, while those with limited access to psychological support may have exhibited greater emotional difficulties. Future research should incorporate these variables to ensure more precise and robust findings.

In addition, the study’s cross-sectional design presents a limitation, as it does not allow for causal inferences. Future research could benefit from longitudinal studies that examine the development of mental health disorders over time and explore potential factors associated with their onset during university years. Additionally, further investigation is recommended into variables such as social support during EA and coping strategies, as these factors may influence academic performance, substance use, and overall mental health.

## Conclusions

This study provides valuable insights into the relationship between drug use, absenteeism, and academic performance among university students. The findings indicate that alcohol, tobacco, and cannabis are the most commonly consumed substances, with usage rates exceeding those reported in national surveys. Importantly, a significant relationship was observed between substance use and lower academic performance, reinforcing previous research that highlights the negative impact of drug consumption on students’ academic outcomes. Additionally, absenteeism emerged as a key predictor of poor academic performance, suggesting that students who miss more classes are at a higher risk of academic difficulties. Beyond substance use, psychological factors such as instability/negativity, depression, and stress were also found to influence academic performance. While instability/negativity and depression were negatively associated with academic success, stress showed a counterintuitive effect, being linked to higher academic performance. This suggests that moderate stress levels may serve as a motivational factor for some students, an area that warrants further investigation. From a practical perspective, these findings can inform university policies and intervention programs to reduce substance use and promote mental well-being among students. Strategies focusing on early detection of risky behaviors, psychological support, and attendance monitoring could be crucial in improving academic outcomes. Moreover, further research should explore the experiences of young people outside the university system, including those who work or are neither studying nor employed, to gain a broader understanding of the factors influencing emerging adulthood.

Addressing substance use and mental health issues within the university setting is essential for fostering a healthier and more academically successful student population. Future interventions should integrate a multidisciplinary approach, considering psychological and behavioral factors to enhance student well-being and academic performance.
